# Necdin regulates BMAL1 stability and circadian clock through SGT1-HSP90 chaperone machinery

**DOI:** 10.1093/nar/gkaa601

**Published:** 2020-07-15

**Authors:** Renbin Lu, Yufan Dong, Jia-Da Li

**Affiliations:** Center for Medical Genetics, School of Life Sciences, Central South University, Changsha 410078, Hunan, P. R. China; Hunan Key Laboratory of Animal Models for Human Diseases, Changsha 410078, Hunan, P. R. China; Center for Medical Genetics, School of Life Sciences, Central South University, Changsha 410078, Hunan, P. R. China; Center for Medical Genetics, School of Life Sciences, Central South University, Changsha 410078, Hunan, P. R. China; Hunan Key Laboratory of Animal Models for Human Diseases, Changsha 410078, Hunan, P. R. China; Hunan Key Laboratory of Medical Genetics, Changsha 410078, Hunan, P. R. China

## Abstract

Circadian clocks are endogenous oscillators that control ∼24-hour physiology and behaviors in virtually all organisms. The circadian oscillator comprises interconnected transcriptional and translational feedback loops, but also requires finely coordinated protein homeostasis including protein degradation and maturation. However, the mechanisms underlying the mammalian clock protein maturation is largely unknown. In this study, we demonstrate that *necdin*, one of the Prader-Willi syndrome (PWS)-causative genes, is highly expressed in the suprachiasmatic nuclei (SCN), the pacemaker of circadian clocks in mammals. Mice deficient in *necdin* show abnormal behaviors during an 8-hour advance jet-lag paradigm and disrupted clock gene expression in the liver. By using yeast two hybrid screening, we identified BMAL1, the core component of the circadian clock, and co-chaperone SGT1 as two necdin-interactive proteins. BMAL1 and SGT1 associated with the N-terminal and C-terminal fragments of necdin, respectively. Mechanistically, necdin enables SGT1-HSP90 chaperone machinery to stabilize BMAL1. Depletion of necdin or SGT1/HSP90 leads to degradation of BMAL1 through the ubiquitin–proteasome system, resulting in alterations in both clock gene expression and circadian rhythms. Taken together, our data identify the PWS-associated protein necdin as a novel regulator of the circadian clock, and further emphasize the critical roles of chaperone machinery in circadian clock regulation.

## INTRODUCTION

Prader–Willi syndrome (PWS) is a genetic disorder induced by a deletion in the paternally-derived chromosome region 15q11–q13, which is a maternally imprinted region. The prevalence of PWS is estimated to be 1/10 000 to 1/25 000 in the general population (1). A number of genes have been mapped within the PWS region, including five protein-coding genes (*MKRN3*, *MAGEL2*, *NDN*, *SNRPN-SNURF*, *C15orf2*) and several antisense transcripts (2,3).

PWS is characterized by feeding problems and failure to thrive in early infancy, followed by growth delay, learning difficulties, hyperphagia and obesity, sleep abnormalities, behavioral problems and hypogonadism (3). Abnormalities in sleep and arousal are among the common features of PWS, which is characterized by excessive daytime sleepiness, defects in organization of rapid eye movement (REM) sleep and arousal, and sleep-disordered breathing (4). But the pathophysiologic mechanisms underlying such sleep disorders in PWS patients remain obscure.

Sleep is regulated by a homeostatic process as well as by circadian rhythms (5,6). A circadian rhythm is an endogenous oscillator that is maintained in an organism's tissues and cells and regulates daily changes in physiology, metabolism and behavior with an approximately 24-h daily period (7,8). The mammalian circadian clock is fundamentally based on a transcriptional-translational feedback loop. At the core of this molecular network are two transcription factors: circadian locomotor output cycles kaput (CLOCK) and brain and muscle aryl hydrocarbon receptor nuclear translocator-like 1 (BMAL1). They heterodimerize and bind to E-box elements (CACGTG) located in the promoters of a large number of clock-controlled genes. This mechanism drives the expression of these genes, including *Period* (*Per1–3*) and *Cryptochrome* (*Cry1/2*). The PER and CRY proteins form a complex and translocate to the nucleus to inhibit the transcriptional activity of the CLOCK-BMAL1 (9,10).

*NDN*, an intronless gene within the PWS region, encodes necdin, a protein with 321 amino acid residues (11). To study the expression pattern of *necdin* in the central nervous system, we searched the Allen Brain Atlas and found that this gene is highly expressed in the suprachiasmatic nucleus (SCN), the pacemaker of the mammalian circadian clock (6,10,12–14), implying that it should have a role in circadian rhythm regulation. In this study, we demonstrate that mice deficient in *necdin* show abnormal behaviors when subjected to an 8-hour advance jet-lag paradigm, accompanied by disrupted expression of clock genes in their livers. Further, we found that necdin interacts with BMAL1 and facilitates SGT1/HSP90 chaperone machinery to stabilize BMAL1. Disruption of *necdin* or *SGT1* expression destabilizes BMAL1 by promoting its proteolytic degradation through the ubiquitin-proteasomal system, resulting in altered clock gene expression and disrupted circadian rhythms. These findings identify a novel mechanism involved in circadian clock regulation and provide evidence implicating necdin as a key endogenous regulator of the circadian clock in mammals.

## MATERIALS AND METHODS

### Sequences for siRNAs

The siRNA sequence of Negative Control was UUCUCC-GAACGUGUCACGUTT. The sense strand sequences of siRNA were CCUGC-ACACCAUGGAGUUUTT (#1), UCAUGAUCCUGAGCCUCAUTT(#2) and GGA-AGAAGCACUCCACCUUTT(#3) for necdin, and GCAGCUUUAAACAGAUUAU-TT(#1), CUGGUAUCAAAC AGAAUCUTT(#2) and GCAGAUGUAAAGAACC-UAUTT(#3) for Sgt1.

### Antibodies and reagents

Antibodies against Necdin (ab18554), SGT1 (ab30931), BMAL1 (ab3350), HSP90 (ab13492), PER1 (ab136451), PER2 (ab180655), CRY1 (ab104736) were purchased from Abcam. Antibody against AVP (sc-390723) were obtained from Santa Cruz. Antibodies against Myc-tag (2276), Flag-tag (14793), HA-tag (3724), VIP (63269) were obtained from Cell Signaling Technology. Antibody against CRY2 were obtained from Invitrogen. Antibody against GRP (20073) were obtained from ImmunoStar. 17-AAG (HY-10211) and Geldanamycin (HY-15230) were purchased from MedChemExpress.

### Animals

*Necdin* mice were generated by using CRISPR-Cas9 technology. Cas9 mRNA and two guide RNAs (gRNA) targeting the upstream and downstream regions of the mouse *necdin* gene were injected into C57BL/6 mouse oocytes, and a mouse with deletion of the entire *necdin* gene was used as a founder. Before behavioral tests, mice of the same sex were group-housed (3–5 animals per cage) under controlled conditions [temperature, 20 ± 2°C; relative humidity, 50–60%; 12:12-h light–dark (LD) cycle, lights on at 7:00 AM and lights off at 7:00 PM] and had free access to food and water. All procedures regarding the care and use of animals were approved by the Institutional Animal Care and Use Committee of Central South University of China.

The primers for genotyping were as following:

Ndn-WT forward (P1): 5′-CTTTCTCCAGG ACCTTCACATTTA-3′
Ndn-WT reverse (P2): 5′-GGGTCGCTCAGGTCCTTACTTTG-3′
Ndn-Mutant forward (P3): 5′-AAACAACTCATCATCATCATAAGG-3′
Ndn-Mutant reverse (P4): 5′-TTTGTAAAGGGTGCTAAGTGC-3′

### Locomotor behavior

Mice aged 4–6 months were individually housed within cages equipped with running wheels and were allowed free access to food and water. Their locomotor activities were recorded as revolutions per 5-min interval. Mice were entrained to an initial LD cycle (light intensity ∼150 lx, lights on at 7:00 AM and lights off at 7:00 PM). After 2–3 weeks of activity recording in 12:12-h light–dark conditions, the mice were placed in constant darkness (DD) or constant light (LL) for ∼4 weeks.

These mice were then subjected to a light-induced phase shift at day 15 of DD. Animals in their home cages were moved to another room and exposed to a 10-min pulse of white light (∼150 lux) at circadian time (CT) 16, at which CT12 was designated as activity onset. The light-induced phase-shift amplitude was derived from regression lines drawn through the activity onset at least 7 days immediately before the day of stimulation and 7 days after reestablishment of a steady-state circadian period after stimulation.

The free-run period and fast Fourier transformation (FFT) were calculated using ClockLab software (Actimetrics, Evanston, IL, USA) in the Matlab environment. The free-run period was measured by a χ^2^ periodogram from days 10 through 25 under DD. Daily revolutions and FFT were determined by analyzing the activity of the last 10 days under LD and days 10–25 under DD. FFT circadian amplitude values represent the peak relative amplitude in the circadian range (18–30 h) normalized to a total variance of 100%.

For the 8-h advance jet-lag experiments, lights were turned off at 11:00 AM and turned on at 11:00 PM on the first day. Thereafter the animals were exposed to the new light–dark schedule for three weeks. The onset of activity for each cycle was defined as the occurrence of the first concentrated bout of activity after an extended period of rest.

### Generation of *Ndn^−p/+m^* fibroblast cells

Briefly, we placed one male *Ndn*^+p/−m^ mouse with two female WT mice in one cage. Once a plug was identified, the female mouse was transferred to a new cage and the time of embryo development was noted as 0.5 days. After the embryos developed for 13.5–15.5 days, the pregnant mouse was euthanized and each embryo was carefully transferred to a fresh dish. The embryos were dissected and the head, heart and liver were removed, with the head being used for genotyping. The fetal tissues were minced and digested with pre-chilled 0.25% trypsin-EDTA at 37°C for 10 min. The suspension was transferred to a 50 ml conical tube and the cells were spun down at 500 × *g* and 4°C for 5 min. Cell pellets from the tube were resuspended in complete culture media and the cells were cultured in a standard humidified 37°C, 5% CO_2_ incubator.

### Cell culture and transfection

U2OS and HEK293T cells were maintained in Dulbecco's modified Eagle's medium (DMEM) supplemented with 10% fetal bovine serum (FBS), 100 units/ml penicillin, and 100 μg/ml streptomycin at 37°C in 5% CO_2_ incubators. Plasmid and siRNA transfections were performed with Lipofectamine 2000 (Invitrogen) reagents according to the manufacturer's protocol.

### Co-immunoprecipitation

Cells were harvested with lysis buffer (150 mM NaCl, 1% NP-40, 2 mM EDTA, 50 mM Tris, pH 8.0, and protease inhibitor cocktail). Approximately 1 mg of whole cell lysate was incubated with 1 μg of the indicated antibody with constant agitation overnight at 4°C. 30 μl protein G agarose bead slurry (Sigma; P3296) was added to pull down the immunocomplexes. The beads were collected by centrifugation, washed extensively with lysis buffer 3–5 times, and boiled with 2× SDS loading buffer. The protein samples were then subjected to immunoblot analysis with the appropriate antibody.

### Immunofluorescence staining

Mice were perfused intracardially with 4% paraformaldehyde, and brains were removed, post-fixed and cryoprotected in 25% sucrose overnight. Eight-micron-thick sections including the SCN region were collected and incubated with primary antibodies to necdin (diluted 1:500), AVP (diluted 1:300), VIP (diluted 1:300), GRP (diluted 1:300) or BMAL1 (diluted 1:200) overnight. The slices were then incubated in the dark with Alexa-conjugated labeled secondary antibodies. Nuclei were stained with DAPI. Slices were imaged using a confocal microscope (TCS SP5; Leica). Fluorescence intensities of BMAL1 were analyzed using fluorescence image analysis software (Lumina Vision, Mitani).

### Ubiquitination assays

Cells were treated with MG132 (10 μM) for 6 h, and then lysed with ubiquitination buffer I (2% SDS, 10 mM Tris-base, pH 7.5, 150 mM NaCl) at 100°C for 10 min. The lysates were then diluted 10-fold with regular ubiquitination buffer II (1% Triton-100, 10 mM Tris-base, pH 7.5, 2 mM EDTA, 150 mM NaCl) and subjected to immunoprecipitation and western blot analysis.

### RNA isolation, reverse transcription and RT-qPCR

Cells or tissues were lysed with TRIzol^®^ reagent according to the manufacturer's instructions. Two micrograms of total RNA were reversed transcribed using RevertAid First Strand cDNA Synthesis Kit (Thermo Scientific; K1622), and 20 ng of total cDNA equivalents were then analyzed using Fast SYBR™ Green Master Mix (Thermo Scientific; 4385612) according to the manufacturer's instructions using a C1000 touch Thermal Cycler. Primers used for qPCR were: mNecdin forward 5′-GAGGTCCCCGACTGTGAGAT-3′ and reverse 5′- TGCAGGATTTTAGGGTCAA-CATC-3′; mBMAL1 forward 5′-TGACCCTCATG-GAAGGTTAGAA-3′ and reverse 5′-GGACATTGCATTGCA-TGTTGG-3′; mPer1 forward 5′-TGAAGCAAGACCG-GGAGAG-3′ and mPer1 reverse 5′-CACACACGCCGTCACATCA-3′; mPer2 forward 5′-GAAAGCTG-TCACCACCATAGAA-3′ and mPer2 reverse 5′-AACTCGCACTTCCTTTTCAGG-3′; mCry1 forward 5′-CACT-GGTTCCGAAAGGGACTC-3′ and mCry1 reverse 5′-CTGAAGCAAAAATC-GCCACCT-3′; mCry2 forward 5′-CACTGGTTCCGCAA-AGGACTA-3′ and mCry2 reverse 5′-CCACGGGTCGAGGATGTAGA-3′; mDbp forward 5′-GGAAACAGCAAGCCCAAAGAA-3′ and mDbp reverse 5′-CAGCGG-CGCAAAAAGACTC-3′; mDec1 forward 5′-ACGGAGACCTGTCAGGGATG-3′ and mDec1 reverse 5′-GGCAGTTTGTAAGTTTCCTTGC-3′; mDec2 forward 5′-TGTGTAAACCCAAAAGGAGCTT-3′ and mDec2 reverse 5′-TGTTCGG-GCAGTAAATCTTTCAG-3′. All reactions were performed in triplicate. The relative levels of gene mRNAs were normalized to the corresponding glyceraldehyde-3-phosphate dehydrogenase (GAPDH) levels.

### Real-time bioluminescence monitoring

U2OS cells stably expressing a luciferase under the control of *Bmal1* promoter were a kind gift from Professor Eric Zhang (NIBS, Chinese Academy of Sciences). Cells were maintained in Dulbecco's modified Eagle's medium (DMEM) supplemented with 10% fetal bovine serum (FBS), 100 units/ml penicillin, 100 ng/ml streptomycin and 5 ng/ml puromycin at 37°C in 5% CO_2_. A total of 6 × 10^5^ cells were plated in 35-mm dishes for 24 h. The cells were treated with 0.1 μM dexamethasone (DEX, Sigma; D4902) for 2 h, and the medium was changed to phenol red-free DMEM supplemented with 10% FBS, 10 mM HEPES buffer, 0.1 mM luciferin (Promega, P1061), 100 units/ml penicillin and 100 ng/ml streptomycin. Bioluminescence was monitored continuously with a 32-channel LumiCycle (Actimetrics, Wilmette, IL, USA) and was analyzed by LumiCycle analysis software (Actimetrics, Wilmette, IL, USA).

### Yeast two-hybrid screening assay

Necdin cDNA was cloned into a pGBKT7 plasmid and used as bait to screen a mouse embryo 11-day library (Clontech/ TaKaRa). Interacting clones were grown on a selection medium lacking the amino acids Leu and Trp and containing Aureobasidin A and X-α-Gal. Auxotrophic marker genes and the β-galactosidase assay were used to screen for positive clones. Plasmids of positive clones were isolated, amplified and sequenced. Interactions were further confirmed by retransforming the identified plasmids together with the bait. The identified DNA sequences were further characterized by BLAST analysis of the NCBI database (http://blast.ncbi.nlm.-nih.gov/Blast.cgi) to determine the identity of potential necdin-interacting proteins.

### Western blotting

Cells or tissue samples were lysed in 2× SDS lysis buffer (2% SDS, 63 mM Tris–HCl, and 10% glycerol). Proteins in lysates were separated by SDS-PAGE, transferred to nitrocellulose membranes (PVDF), and immunoblotted with the corresponding antibodies overnight at 4°C. Membranes were then washed and incubated with horseradish peroxidase conjugate secondary antibodies. The proteins were visualized using the Pierce™ ECL Western Blotting Substrate kit (Thermo Scientific; 32106). Band intensities were quantified by ImageJ.

### Statistical analysis

Statistical analyses were performed using GraphPad Prism 6.0. All experiments were repeated at least three times and the distribution of data points is presented as mean ± s.e.m. Student's *t*-test for comparison of two conditions or ANOVAs were utilized with *post hoc* Bonferroni multiple-comparisons test for three or more conditions. *P* < 0.05 was considered significant.

## RESULTS

### Circadian behaviors and clock gene expression are altered in *necdin*-deficient mice

**Figure 1.**
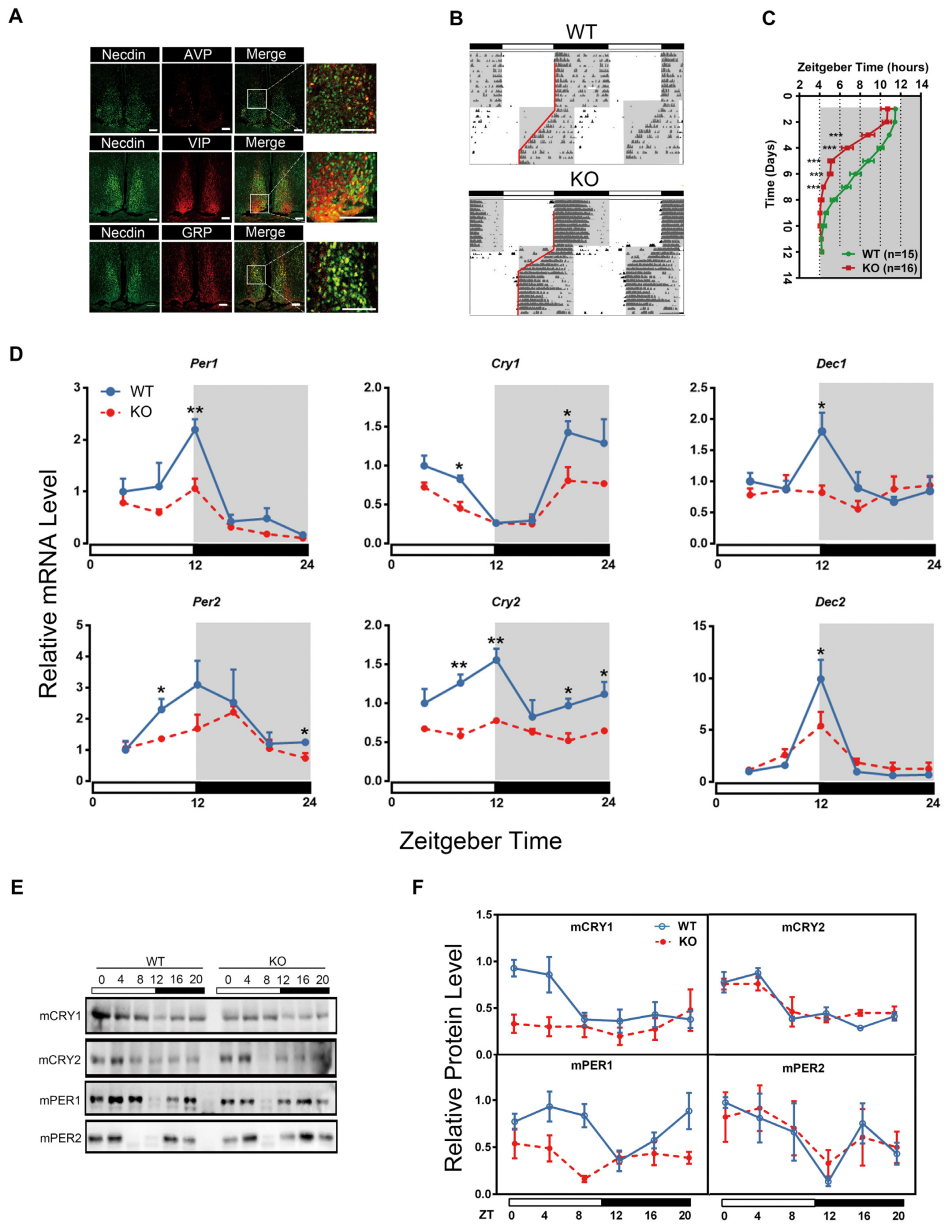
Altered circadian behaviors and clock gene expression in necdin-deficient mice. (**A**) Expression of necdin (green), AVP (red), VIP and GRP (red) in the SCN using immunofluorescence staining. Scale bar, 100 μm. (**B**) Representative double-plotted actograms of wild-type (WT) and *necdin* KO mice subjected to an 8-h advanced jet-lag paradigm. The major activity onset during shift is labelled with red lines. (**C**) Statistics data of major activity onset plot from WT (*n* = 15) and *necdin* KO mice (*n* = 16). Data are presented as means ± s.e.m.; *P < 0.05; **P < 0.01; ***P < 0.005, Bonferroni *post hoc* analysis, two-way ANOVA. **(D)** Quantitative PCR analysis of the indicated clock genes in the livers from WT and *Necdin* KO mice taken at indicated ZTs of a day. Data are presented as means ± s.e.m.; *P < 0.05; **P < 0.01; ***P < 0.005, Bonferroni *post hoc* analysis, two-way ANOVA; *n* = 3 mice/genotype/time point. (**E**, **F**) Western blot analysis of liver nuclear extracts from WT and *necdin* KO mice over a 24 h circadian cycle (E) and the quantification of indicated protein levels (F); Data are presented as means ± s.e.m.; *P < 0.05; **P < 0.01; ***P < 0.005, Bonferroni *post hoc* analysis, two-way ANOVA; *n* = 3 mice/genotype/time point.

The SCN located in the hypothalamus is the pacemaker of the circadian clock in mammals. Using immunofluorescence staining, we found that necdin is highly expressed in the SCN (Supplementary Figure S1) and partly co-localizes with several SCN neuropeptides including arginine vasopressin (AVP), vasoactive intestinal polypeptide (VIP), gastrin releasing peptide (GRP) (Figure 1A). Nevertheless, necdin protein levels in the SCN were unchanged between the day and night (data not shown).

To elucidate the physiological function of necdin, we generated a mouse strain with a *necdin*-null allele (Supplementary Figure S2A and B). As *necdin* is a maternally imprinted gene, mice with defects in paternal *necdin* (*necdin*^−p/+m^) lack *necdin* expression (Supplementary Figure S2C and D). To address the function of necdin in circadian regulation, we monitored the wheel-running activity of *necdin*^−p/+m^ mice and their wild-type (WT) littermate controls. Animals were housed individually in cages equipped with running wheels. After continuous monitoring of wheel running activities for 2–3 weeks under a 12:12-h light–dark (LD) schedule, mice were switched to constant darkness (DD) or constant light (LL) for 4 wk. Both WT and *necdin*^−p/+m^ mice entrained to LD cycles and showed no significant differences in daily counts or amplitudes of locomotor rhythmicity (Supplementary Figure S3A). Under DD or LL conditions, both WT and *necdin*^−p/+m^ mice showed similar free-running periods and fast Fourier transform (FFT) power levels (Supplementary Figure S3B, C and E). We also compared the phase shifts generated by exposure to a brief light pulse at CT16 (white light, ∼150 lux, 10 min) under DD conditions. In response to this treatment, we did not see any significant difference between WT and *necdin*^−p/+m^ mice (WT, 121.0 ± 21.3 min, *n* = 6; *necdin*^−p/+m^, 113.7 ± 1.7 min, *n* = 6; *P*> 0.05) (Supplementary Figures S3A and S3D).

We then compared the adaptation of WT and *necdin*^−p/+m^ mice to an 8-h advance jet-lag paradigm. WT mice gradually shifted their locomotor activity rhythms to the new LD schedule. Every subsequent day after the LD cycle advance, WT mice started their activity slightly earlier, and finally aligned it to the beginning of night after ∼11 days. In contrast, *necdin*^−p/+m^ mice adapted more rapidly to the 8-h advance, requiring only ∼8 days for re-entrainment (Figure 1B, C). There was a significant difference in PS50 (50% phase-shift) values between the two strains (WT, 5.89 ± 0.11 days, *n* = 15; *necdin*^−p/+m^, 3.71 ± 0.12 days, *n* = 16; *P* < 0.05, Figure 1C). Although most *necdin*^−p/+m^ mice showed a gradual shift to the new LD cycle, activity bouts clearly occurred between lights-off and the major activity onset during the shift; such bouts were absent in WT mice. Furthermore, Jet-lag treatment disrupted the behaviors in another two *necdin*^−p/+m^ mice (Supplementary Figure S3F), implying an unstable central clock in these mice.

We also measured the expression levels of a panel of clock genes in the livers of WT and *necdin*^−p/+m^ mice at different time points during a circadian cycle. As shown in Figure 1D, expression of the clock genes *Per1/2*, *Cry1/2* and *Dec1/2* showed a definite circadian rhythm in WT mice, the amplitude of which was significantly attenuated in *necdin*^−p/+m^ mice. Necdin deficiency also led to significantly damped circadian amplitude of mCRY1 and mPER1 proteins in the liver nuclear extracts, whereas the patterns of mCRY2 and mPER2 in the liver nuclear extracts were largely intact (Figure 1E, F). Our data thus demonstrate an important role of necdin in the circadian regulation in mice.

### Necdin interacts with BMAL1

**Figure 2.**
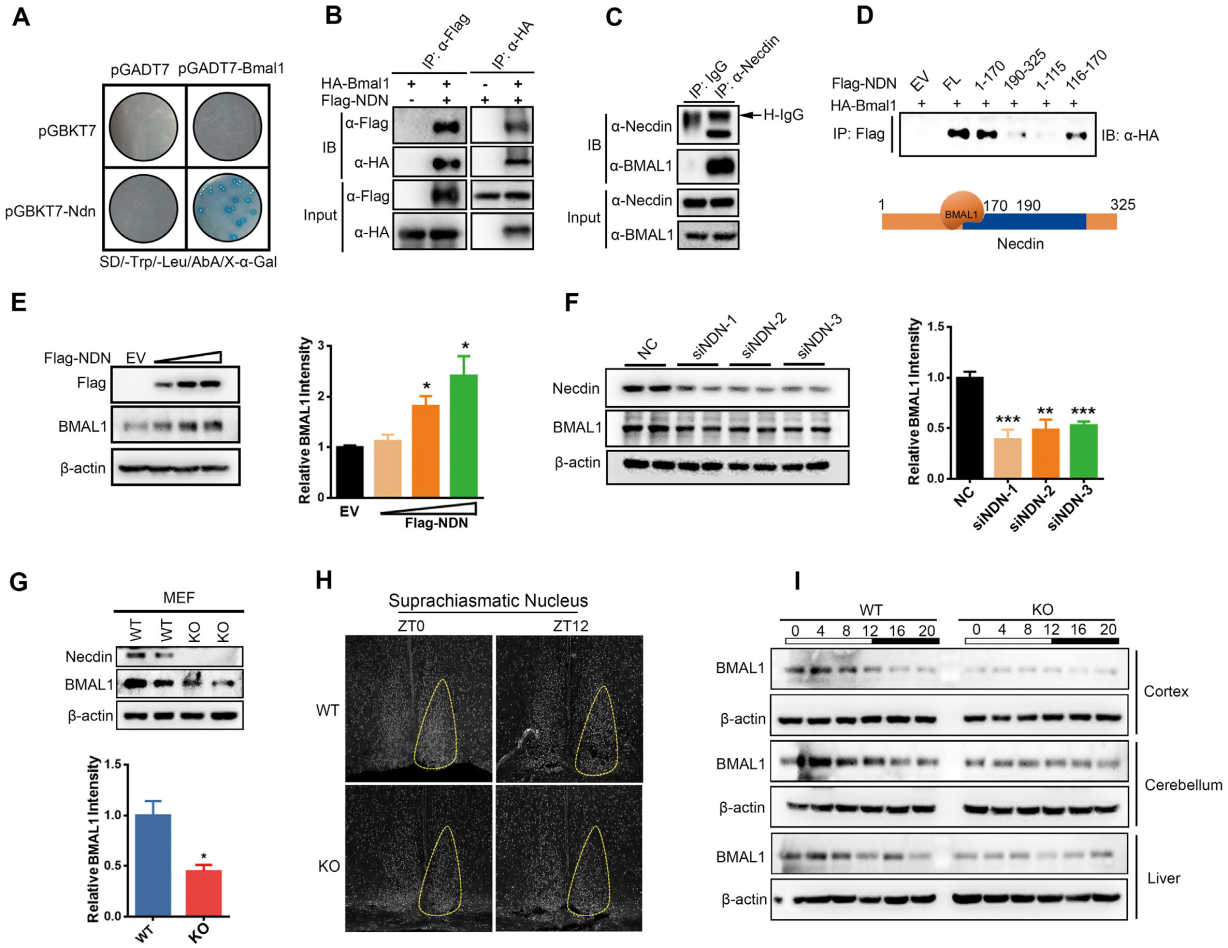
Necdin positively regulates BMAL1 protein levels. (**A**) Interaction of necdin and BMAL1 as assayed with a yeast two hybrid assay. The term pGBKT7-Ndn denotes necdin fused to a DNA-binding domain, whereas pGADT7-Bmal1 denotes BMAL1 fused to an activation domain. Only yeast colonies co-transformed with pGADT7-Bmal1 and pGBKT7-Ndn can grow and turn blue on a SD/-Leu/-Trp/AbA/X-α-Gal plate. (**B**) Interaction of necdin and BMAL1 as assayed by a co-immunoprecipitation (co-IP) assay in HEK293T cells expressing FLAG-necdin and HA-BMAL1. Antibodies against HA, FLAG were used to immunoprecipitate (IP) the cell lysate, and the immune complex was blotted with antibody against HA or FLAG, respectively. (**C**) Interaction of necdin and BMAL1 as assayed by a co-IP assay in U2OS cells. The cell lysate was immunoprecipitated with an antibody against necdin or control IgG, and the immune complex was blotted with antibodies against necdin or BMAL1, respectively. (**D**) Interaction of BMAL1 and full length or truncated necdin as assayed by a co-IP assay. HA-tagged BMAL1 and FLAG-tagged necdin fragments were expressed in HEK293T cells; the cell lysate was immunoprecipitated with FLAG antibody, and the immune complex was blotted with HA antibody. (**E**) Representative immunoblots (left) and statistics data of three independent experiments (right) from U2OS cells transfected with different doses of necdin or empty vector (EV). Data are presented as means ± s.e.m., *P < 0.05, post hoc Dunnett's *t*-test, one-way ANOVA. (**F**) Representative immunoblots (left) and statistics data of three independent experiments (right) from U2OS cells transfected with control siRNA (NC) or NDN siRNAs. Data are presented as means ± s.e.m.; *P < 0.05, *post hoc* Dunnett's *t*-test, one-way ANOVA. (**G**) Representative immunoblots (top) and statistics data of three independent experiments (bottom) from WT and necdin KO MEF cells. Data are presented as means ± s.e.m.; *P < 0.05, unpaired *t-*test. (**H**). Representative BMAL1 immunofluorescence in the SCN from WT and necdin KO mice taken at ZT0 and ZT12. (**I**) Immunoblots of BMAL1 and β-actin in the cortex, cerebellum, and liver from WT and necdin KO mice taken at the indicated ZTs of a day.

To study the underlying mechanism by which necdin affects the circadian rhythm, we performed a yeast two hybrid screen using necdin as bait. As a result, BMAL1, a core component of the circadian clock, was identified as one of the necdin-interactive proteins. As shown in Figure 2A, only yeast colonies co-transformed with both pGBKT-necdin and pGADT7-Bmal1 plasmids grew and became blue on high-stringency plates (SD/-Trp/-Leu/AbA/ X-α-Gal), whereas no colonies grew when yeast was transformed with pGBKT7-necdin and pGADT7, or pGBKT7 and pGADT7-Bmal1.

We further studied the necdin-BMAL1 interaction by using coimmunoprecipitation (co-IP). Only when HA-tagged BMAL1 and FLAG-tagged necdin were co-expressed in HEK293T cells, the FLAG antibody was able to pulldown HA-tagged BMAL1 and the HA antibody was able to pulldown FLAG-tagged necdin (Figure 2B). We also performed co-IP in U2OS cells with an antibody against necdin or control IgG. As shown in Figure 2C, BMAL1 was detected in the immunocomplex pulled down by necdin antibody, but not by control IgG, indicating that necdin interacts with BMAL1 endogenously.

To determine which domains in necdin might be involved in the interaction with BMAL1, we carried out co-IP using full length necdin or several truncated necdin fragments (amino acids 1–115, 1–170, 116–170, 190–325). As shown in Figure 2D, only full length and two N-terminal fragments (amino acids 1–170, 116–170) associated with BMAL1.

### Necdin positively regulates BMAL1 protein levels

To study the functional consequences of the necdin-BMAL1 interaction, we first overexpressed different amounts of necdin in U2OS cells and measured the endogenous BMAL1 protein levels. As shown in Figure 2E, necdin dose-dependently increased endogenous BMAL1 protein levels. Conversely, knockdown of necdin with siRNAs significantly downregulated BMAL1 (Figure 2F). Moreover, BMAL1 protein levels were also significantly decreased in embryonic fibroblasts (MEFs) from *Ndn*^−p/+m^ mice as compared with WT controls (Figure 2G), and the decrease was rescued by overexpression of necdin (Supplementary Figure S4A).

We also measured BMAL1 protein levels in a variety of tissues from WT and *Ndn*^−p/+m^ mice. As shown in Figure 2H, BMAL1 levels in the SCN of *Ndn*^−p/+m^ mice were significantly decreased as compared to WT controls as measured by immunofluorescence staining. Further, BMAL1 protein levels showed a circadian rhythm in the cortex (peak at ZT4, trough at ZT12, ratio of peak to trough was 1.9), cerebellum (peak at ZT0, trough at ZT16, ratio of peak to trough was 2) and liver (peak at ZT8, trough at ZT20, ratio of peak to trough was 2.7), which was significantly attenuated in *Ndn*^−p/+m^ mice (Figure 2I and Supplementary Figure S5). Taken together, these data indicate that necdin positively regulates BMAL1 protein levels.

### Depletion of necdin promotes BMAL1 degradation through the ubiquitin-proteasomal system

**Figure 3.**
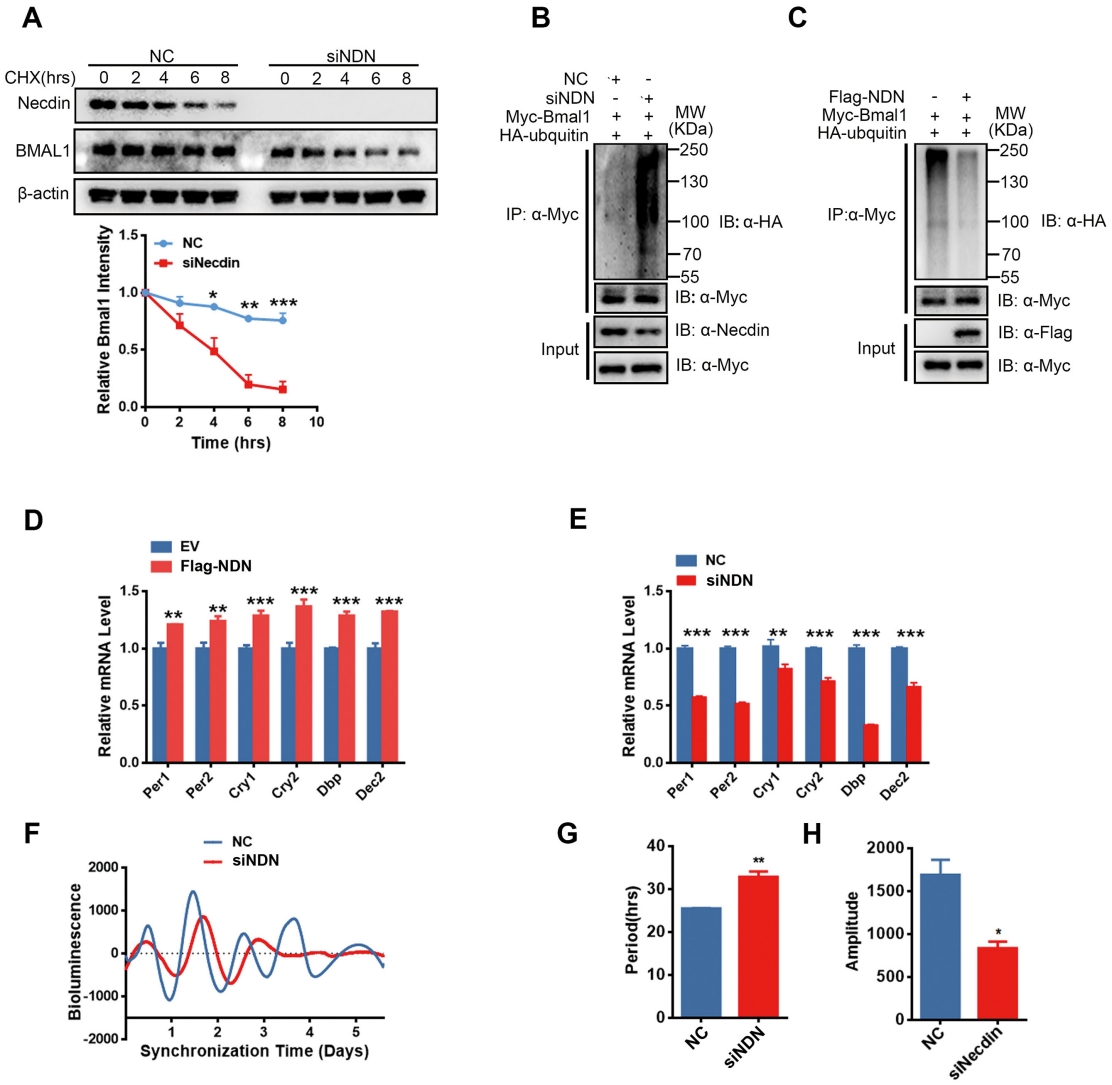
Depletion of necdin promotes BMAL1 degradation through the ubiquitin proteasomal system. (**A**) Representative immunoblots (top) and statistics data of three independent experiments (bottom) from U2OS cells transfected with control siRNA (NC) or NDN siRNA and treated with 10 mg/ml cycloheximide (CHX) for 0–8 h. Data are presented as means ± s.e.m.; *P < 0.05; **P < 0.01; ***P < 0.005, Bonferroni *post hoc* analysis, two-way ANOVA. (**B**) Ubiquitination of BMAL1 in U2OS cells transfected with control siRNA (NC) or NDN siRNA. (**C**) Ubiquitination of BMAL1 in U2OS cells transfected with empty vector or *necdin*. (**D**, **E**) Quantitative PCR of the indicated clock genes in U2OS cells transfected with empty vector (EV) or *necdin* (D), or control siRNA (NC) or NDN siRNA (E). Data are presented as means ± s.e.m.; *P < 0.05; **P < 0.01; ***P < 0.005, Student's *t*-test, *n* = 3. (**F**) Representative bioluminescence signals from control (NC) or NDN-depleted (siNDN) U2OS cells stably expressing luciferase under the control of *Bmal1* promoter. Cells were synchronized with 100 nM dexamethasone and recorded in a Luminometer. (**G**, **H**) Statistics data of the amplitudes (G) and periods (H) obtained from Bmal1:luciferase U2OS cells transfected with control siRNA (NC) or NDN siRNA. Data are presented as means ± s.e.m.; *P < 0.05; **P < 0.01; ***P < 0.005, Student's *t-*test, *n* = 6.

As overexpression or knockdown of necdin did not affect *Bmal1* transcription (Supplementary Figure S6A, B), we speculated that necdin may regulate BMAL1 protein stability. Indeed, knockdown of necdin significantly decreased the half-life of BMAL1 (Figure 3A). Furthermore, overexpression of necdin markedly decreased, whereas knockdown of necdin increased the levels of ubiquitinylated BMAL1 (Figure 3B, C), indicating that depletion of necdin destabilizes BMAL1 through the ubiquitin-proteasomal system.

BMAL1 is one of the core components of the circadian clock. BMAL1 and CLOCK form a heterodimer that positively regulates the transcription of downstream genes through binding to the E-box elements on target gene promoters. We therefore measured the expression of several BMAL1-CLOCK target genes after overexpression or depletion of necdin in U2OS cells. As shown in Figure 3D and Figure 3E, overexpression of necdin increased, whereas depletion of necdin decreased the transcription of *Per1/2*, *Cry1/2*, *Dbp* and *Dec2* in U2OS cells. We also measured the circadian rhythm of U2OS cells stably expressing luciferase under control of the *Bmal1* promoter. Depletion of necdin significantly decreased the circadian amplitude (Control siRNA, 1690 ± 175.0; *Necdin* siRNA, 834.9 ± 76.1; *P* < 0.05; Figure 3F, G) and lengthened the circadian period (Control siRNA, 25.50 ± 0.06; *Necdin* siRNA, 32.83 ± 1.3; *P* < 0.01; Figure 3H).

### Necdin bridges BMAL1 and SGT1 to form a complex

SGT1 is another necdin-interactive protein identified from the above-mentioned yeast two hybrid screening (Supplementary Figure S7A). We chose SGT1 for further study as SGT1 is a co-chaperone of HSP90, a protein which facilitates the maturation and stabilization of a wide range of client proteins and is a well-known regulator of the circadian clock in mammals, *Arabidopsis* and *Drosophila* (15–17).

**Figure 4.**
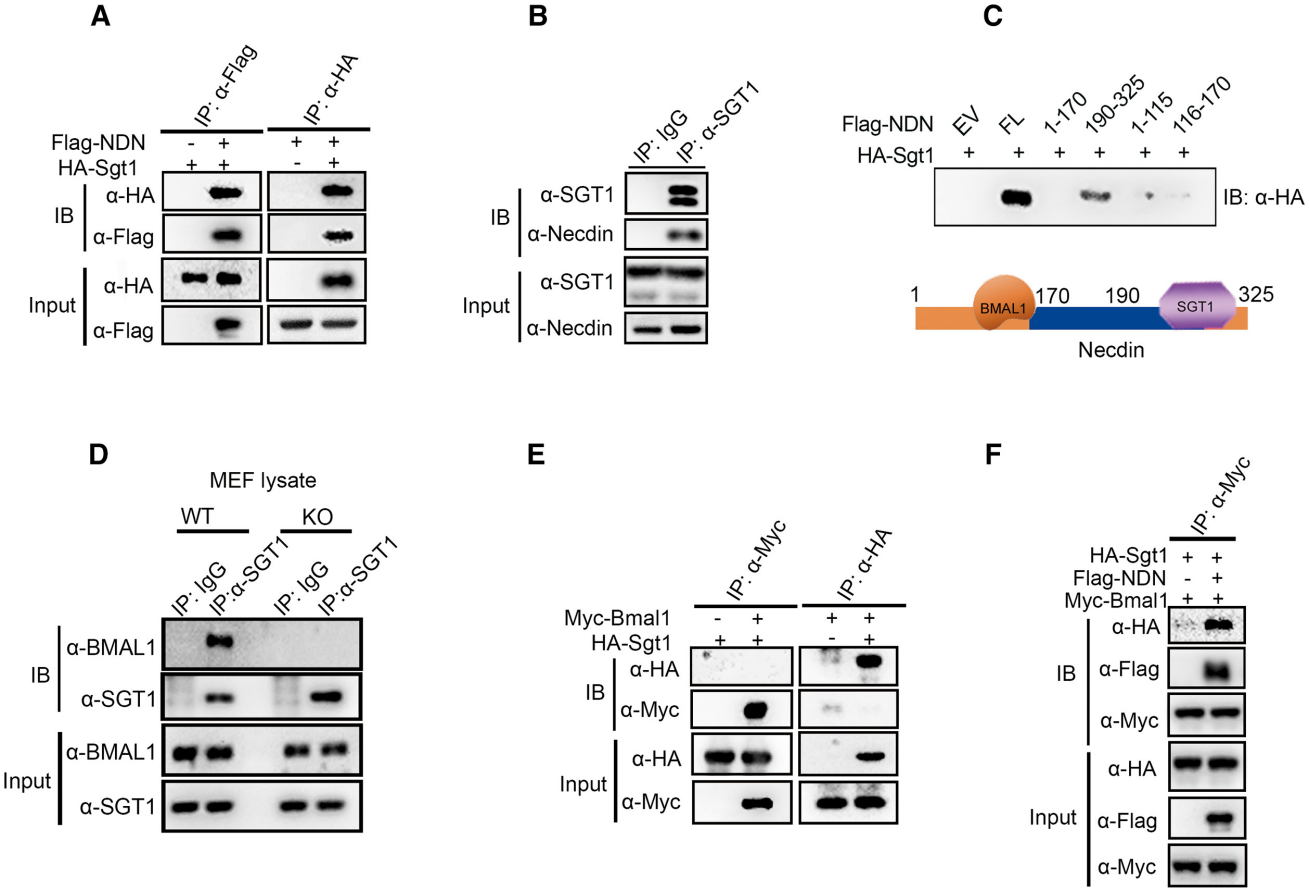
Necdin bridges BMAL1 to form a complex with SGT1. (**A**) Interaction of necdin and SGT1 as assayed by co-immunoprecipitation (co-IP) assay in HEK293T cells expressing FLAG-necdin and HA-SGT1. Antibodies against HA, FLAG were used to immunoprecipitate (IP) cell lysate, and the immune complex was blotted with antibody against HA or FLAG, respectively. (**B**) Interaction of necdin and BMAL1 as assayed by co-IP assay in U2OS cells. Cell lysate was immunoprecipitated with an antibody against SGT1 or control IgG, and the immune complex was blotted with antibodies against necdin or SGT1, respectively. (**C**) Interaction of BMAL1 and full length or truncated necdin as assayed by co-IP assay. HA-tagged SGT1 and FLAG-tagged necdin fragments were expressed in HEK293T cells, cell lysate was immunoprecipitated with FLAG antibody, and the immune-complex was blotted with HA antibody. A diagram indicating the interaction of necdin with BMAL1 and SGT1 is shown at the bottom. (**D**) An anti-necdin antibody was used to immunoprecipitate the cell lysate from WT and necdin-KO MEFs, and the immune complexes were blotted with the indicated antibodies. (**E**) Co-IP assay of HEK293T cells expressing Myc-BMAL1 and HA-SGT1. Antibodies against HA, Myc or control IgG were used to immunoprecipitate (IP) cell lysate, and the immune complex was blotted with antibody against HA or Myc, respectively. (**F**) An anti-Myc antibody or control IgG was used to immunoprecipitate cell lysate from HEK293T cells expressing FLAG-necdin, Myc-BMAL1 or HA-SGT1; immune-complexes were blotted with the indicated antibodies.

We confirmed the interaction between necdin and SGT1 by a co-IP assay. Only when HA-tagged SGT1 and FLAG-tagged necdin were co-expressed in HEK293T cells, the FLAG antibody was able to pulldown HA-tagged SGT1 and the HA antibody was able to pulldown FLAG-tagged necdin (Figure 4A). We also performed co-IP in U2OS cells with an antibody against SGT1 or control IgG. As shown in Figure 4B, necdin was detected in the immunocomplex pulled-down by SGT1 antibody, but not by control IgG, indicating that necdin interacts with SGT1 endogenously.

SGT1 bound to full-length necdin or to a C-terminal fragment (amino acids 190–325) of necdin in the co-IP assay (Figure 4C). We therefore speculate that necdin may bridge BMAL1 and SGT1 to form a complex (Figure 4C, bottom). Indeed, antibody against SGT1 was able to pull down BMAL1 in U2OS cells (Supplementary Figure S7B) and in WT MEF cells, but not in *Ndn*^−p/+m^ MEF cells (Figure 4D). When HA-SGT1 and Myc-BMAL1 were co-expressed in HEK293T cells, HA antibody failed to pull down Myc-BMAL1 (Figure 4E). Similarly, Myc antibody was unable to pull down HA-SGT1 (Figure 4E). Yet, HA antibody pulled down Myc-BMAL1 when Myc-BMAL1, HA-SGT1 and FLAG-necdin were co-expressed in HEK293T cells (Figure 4F). These results indicate that necdin was required and sufficient for formation of a complex between it, BMAL1 and SGT1.

### SGT1 regulates BMAL1 stability in a necdin-dependent manner

**Figure 5.**
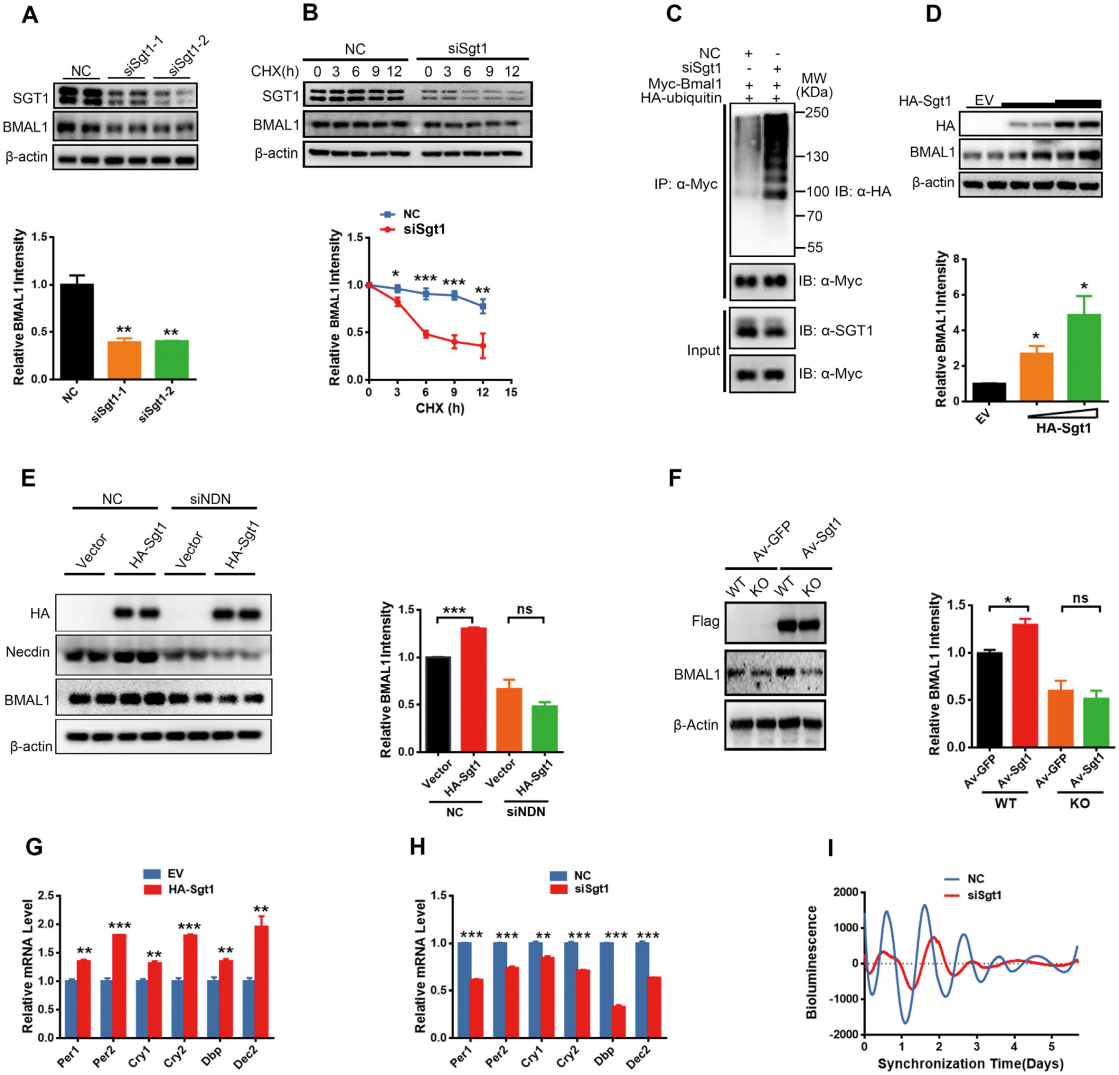
SGT1 regulates BMAL1 stability in a necdin-dependent manner. (**A**) The representative immunoblots (top) and statistics data of three independent experiments (bottom) from U2OS cells transfected with control siRNA (NC) or Sgt1 siRNAs. Data are presented as means ± s.e.m.; **P < 0.01, post hoc Dunnett's *t*-test, one-way ANOVA. (**B**) Representative immunoblots (top) and statistics data of three independent experiments (bottom) from U2OS cells transfected with control siRNA (NC) or Sgt1 siRNA and treated with 10 mg/ml CHX for 0–12 h. Data are presented as means ± s.e.m.; *P < 0.05; **P < 0.01; ***P < 0.005, Bonferroni *post hoc* analysis, two-way ANOVA. (**C**) The ubiquitination of BMAL1 in U2OS cells transfected with control siRNA (NC) or Sgt1 siRNA. (**D**) Representative immunoblots (top) and statistics data of three independent experiments (bottom) from U2OS cells transfected with different doses of NDN or empty vector (EV). Data are presented as means ± s.e.m.; *P < 0.05, *post hoc* Dunnett's *t*-test, one-way ANOVA. (**E**) SGT1 failed to increase the BMAL1 protein level in necdin-depleted (siNDN) U2OS cells. Representative immunoblots are shown at left; statistics data of three independent experiments are shown at right. Data are presented as means ± s.e.m.; *P < 0.05, unpaired *t-*test. (**F**) SGT1 failed to increase BMAL1 protein levels in *necdin*-KO MEF cells. Representative immunoblots are shown at left; statistics data of three independent experiments are shown at right. Data are presented as means ± s.e.m.; *P < 0.05, unpaired *t-*test. (**G**, **H**) Quantitative PCR of the indicated clock genes in U2OS cells transfected with empty vector (EV) or SGT (G), or control siRNA (NC) or *NDN* siRNA (H). Data are presented as means ± s.e.m.; *P < 0.05; **P < 0.01; ***P < 0.005, Student's *t-*test, *n* = 3. (**I**) Representative bioluminescence signals from control (NC) or Sgt1-depleted (siSgt1) U2OS cells stably expressing luciferase under the control of the *Bmal1* promoter. Cells were synchronized with 100 nM dexamethasone and luminescence was recorded by a Luminometer.

To investigate the effect of SGT1 on BMAL1, we knocked down SGT1 by siRNA in U2OS cells and measured the endogenous BMAL1 protein levels. As shown in Figure 5A, depletion of SGT1 significantly decreased BMAL1 protein levels. Moreover, knockdown of SGT1 decreased the half-life of BMAL1 (Figure 5B) and increased ubiquitination of BMAL1 (Figure 5C). Conversely, overexpression of SGT1 dose-dependently increased BMAL1 protein levels (Figure 5D). Nevertheless, necdin is required for SGT1 to stabilize BMAL1, as overexpression of SGT1 failed to increase the BMAL1 protein level in necdin-depleted U2OS cells (Figure 5E) or *Ndn*^−p/+m^ MEF cells (Figure 5F).

As expected, overexpression of SGT1 increased, whereas depletion of Sgt1 decreased the transcription of *Per1/2*, *Cry1/2*, *Dbp* and *Dec2* in U2OS cells (Figure 5G, H). Depletion of SGT1 in U2OS cells significantly decreased its circadian amplitude (Control siRNA, 2062 ± 184.93; *Sgt1* siRNA, 920.3 ± 35.80; *P* < 0.05; Figure 5I and Supplementary Figure S8A) and lengthened its circadian period (Control siRNA, 24.17 ± 0.52; *Sgt1* siRNA, 26.70 ± 0.35, *P* < 0.05; Figure 5I and Supplementary Figure S8B).

### Necdin regulates BMAL1 stability in an SGT1- and HSP90-dependent manner

**Figure 6.**
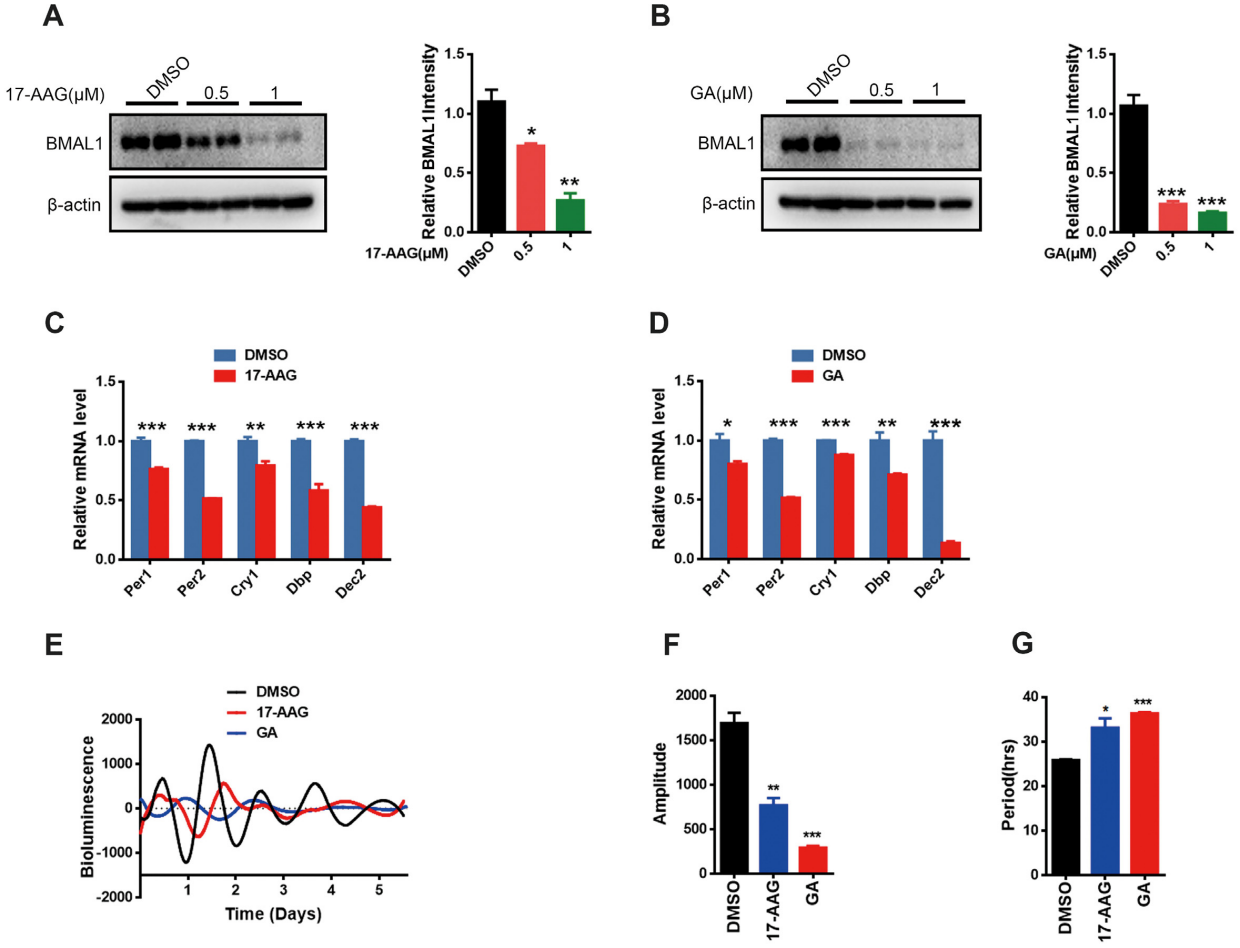
HSP90 regulates BMAL1 stability and clock gene expression. (**A**, **B**) Representative immunoblots (left) and statistics data of three independent experiments (right) from U2OS cells following treatment with different concentrations of 17-AAG (A) or GA (B). Data are presented as means ± s.e.m.; *P < 0.05; **P < 0.01, *post hoc* Dunnett's *t*-test, one way ANOVA. (**C**, **D**) Quantitative PCR of the indicated clock genes in U2OS cells following treatment with 17-AAG (C) or GA (D). Data are presented as means ± s.e.m.; *P < 0.05; **P < 0.01; ***P < 0.005, Student's *t* test, *n* = 3. (**E**) Representative bioluminescence signals from control (DMSO)-, 17-AAG- or GA-treated U2OS cells stably expressing luciferase under control of the *Bmal1* promoter. Cells were synchronized with 100 nM dexamethasone and recorded by a Luminometer. (**F**, **G**) Statistics data of the amplitudes (F) and periods (G) obtained from Bmal1:luciferase U2OS cells treated with DMSO, 17-AAG or GA. Data are presented as means ± s.e.m.; *P < 0.05; **P < 0.01; ***P < 0.005, *post hoc* Dunnett's *t*-test, one way ANOVA. *n* = 6.

**Figure 7.**
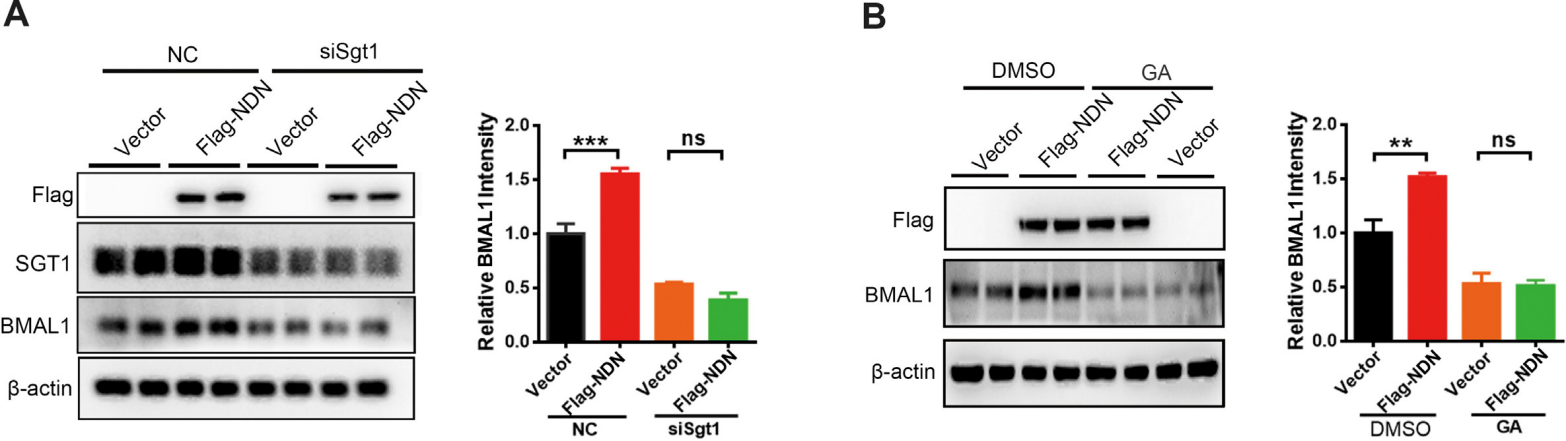
Necdin regulates BMAL1 stability in a SGT1- and HSP90-dependent manner (**A**) Necdin failed to increase BMAL1 protein levels in Sgt1-depleted (siSgt1) U2OS cells. Representative immunoblots are shown at top; statistics data of three independent experiments are shown at bottom; data are presented as means ± s.e.m.; *P < 0.05, unpaired *t* test. (**B**) Necdin failed to increase BMAL1 protein levels in U2OS cells treated with geldanamycin (GA). Representative immunoblots are shown at top; statistics data of three independent experiments are shown at bottom; data are presented as means ± s.e.m.; *P < 0.05, unpaired *t-*test.

SGT1 is a co-chaperone for heat shock protein 90 (HSP90); among its effects, Schneider *et al.* have shown that HSP90 can regulate the stability of BMAL1 and circadian gene expression. Consistent with their data, HSP90 inhibitors 17-AAG and geldanamycin (GA) downregulated BMAL1 protein levels (Figure 6A, B). Furthermore, treatment with HSP90 inhibitors 17-AAG or GA decreased the transcription of *Per1/2*, *Cry1*, *Dbp*, and *Dec2* in U2OS cells (Figure 6C, D). Moreover, treatment with 17-AAG or GA decreased the amplitude of the circadian rhythm (DMSO, 1691 ± 115.9; 17-AAG, 767.2 ± 83.10; GA, 293.7 ± 20.95; *P* < 0.05; Figure 6E and Figure 6F), and increased the circadian period (DMSO, 25.83 ± 0.20 h; 17-AAG, 33.10 ± 2.14 h; GA, 36.37 ± 0.23 h; *P* < 0.05; Figure 6E and G). Interestingly, overexpression of necdin in SGT1-depleted U2OS cells failed to increase BMAL1 protein levels (Figure 7A), indicating that SGT1 is required for necdin to stabilize BMAL1 protein. Furthermore, overexpression of necdin in GA-treated U2OS cells failed to increase the BMAL1 protein level (Figure 7B), indicating the requirement of HSP90 for necdin to stabilize BMAL1 protein.

## DISCUSSION

### Roles of PWS-associated proteins in the circadian clock

PWS-related sleep problems may arise from a variety of mechanisms. Defects in REM sleep may be due to disrupted sleep homeostatic regulation, whereas sleep-disordered breathing may be a consequence of obesity (18). REM sleep defects and/or sleep-disordered breathing may lead to excessive daytime sleepiness (19), which may also be attributable to dysfunctional circadian clocks.

*Magel2* is the first PWS-associated gene that is reported to be involved in sleep regulation. Kozlov *et al.* found that mice deficient in Magel2 showed markedly reduced circadian amplitude of activity and increased daytime activity. They further showed that orexin, a critical wake-promoting protein, is substantially reduced in the lateral hypothalamus of Magel2-deficient mice (20). These data indicate that Magel2 deficiency may be responsible for, at least partly, the sleep homeostatic dysregulation in PWS patients. Nevertheless, Devos *et al.* found that Magel2 is able to repress the activity of the CLOCK-BMAL1 heterodimer in a Per2-luciferase assay, implying a potential role in circadian regulation (21).

Magel2 and necdin belong to the so-called MAGE protein family (22). Interestingly, Xu and colleagues showed that mice lacking MAGED1, a MAGE family member, leads to a shortened period and altered rest-activity bouts. They further identified MAGED1 as an RORα-binding protein that regulates the expression of core clock genes such as *Bmal1*, *Rev-erbα* and *E4bp4* (23).

In this study, we showed that the PWS-associated protein necdin interacts with the core circadian component BMAL1. Necdin stabilized BMAL1 through SGT1-HSP90 chaperone machinery. Deficiency in *necdin* led to altered clock gene expression and circadian behavior, which may underlie part of the circadian dysfunction in PWS patients.

### The roles of HSP90 and protein quality control in circadian clocks

In addition to the interconnected transcriptional and translational feedback loops, the circadian clock is also regulated by numerous post-transcriptional and post-translational processes. The protein homeostasis process, involving the synthesis, folding, maturation, and turnover of polypeptides, has been recognized as being essential for circadian regulation (24).

To prevent mis-folding and facilitate folding, a wide range of molecular chaperones function in cells to stabilize and/or help a non-native protein acquire its native conformation (25,26). In particular, the HSP90-related protein quality control pathway has been reported to participate in circadian control in flies, plants and mammals (15–17).

HSP90 functions downstream of the ribosome-bound HSP70 in the structural maturation and conformational regulation of numerous molecules (27). In *Arabidopsis*, HSP90 stabilizes the circadian clock-associated F-box protein ZEITLUPE. Further studies indicate that GIGANTEA acts as a co-chaperone of HSP90 to facilitate the maturation of ZEITLUPE (28). In *Drosophila*, pharmacologically or genetically reduced HSP90 levels increase behavioral variability of circadian locomotor activity (17).

In mammals, Schneider *et al.* indicated that inhibition of the ATP-dependent chaperone activity of HSP90 impairs circadian rhythmicity of cultured mouse fibroblasts. Inhibition of HSP90 shortened the half-life of BMAL1 and blunted expression of rhythmic BMAL1-CLOCK target genes (15).

In this study, we found that necdin may enable SGT1-HSP90 chaperone machinery to stabilize BMAL1. Although necdin levels did not show circadian variation in the SCN or liver, we speculate that the circadian rhythm of BMAL1 protein levels may be controlled in part by the oscillation of HSP90, as levels of two forms of HSP90 (HSP86 and HSP84) in the mouse SCN displayed an overt circadian rhythm with a peak at night and a trough during the day (29).

### The mechanism underlying how necdin affects protein stability and function

Necdin is involved in a variety of physiological functions, including, adipogenesis, myogenesis as well as neuronal differentiation, survival and migration (30,31). Deficiency in necdin leads to hypothalamic dysfunction and central apnea, which mimic some symptoms of' PWS (32).

Necdin may regulate its target genes/proteins at either the transcriptional or post-translational levels. Although necdin has no transcriptional activity, it may interact with transcription activators/repressors to regulate transcription indirectly. Necdin may enhance target gene expression by relieving the repression of some transcriptional suppressors, such as Bmi1, Msx and EID-1 (33–35). Necdin may also down-regulate gene expression by suppressing the activity of certain transactivators, such as p53, Sp1, E2F1, ARNT2:SIM1 and ARNT2:HIF1α (36–39). Furthermore, necdin has been shown to regulate PPARγ expression through an epigenetic mechanism, by affecting both the recruitment of MeCP2 (methyl CpG binding protein 2) and HP-1α co-repressor to the *Pparγ* promoter as well as the H3K27 dimethylation status at the exon 5 locus (40).

Necdin may antagonize transcription repressors or activators at the post-translational level. Yoshikawa and colleagues found that necdin is able to recruit Sirt1 and consequently facilitate the de-acetylation of Foxo1 or p53, leading to diminished transcription activity of these two factors (41,42).

Necdin may also regulate the ubiquitination and stability of proteins with which it interacts. In this scenario, necdin stabilizes PGC-1α and leptin receptor, but promotes the degradation of HIF-1α, PIAS1 and CCAR1 (43–46). Although the underlying mechanism is largely unknown, Wijesuriya *et al.* demonstrated that necdin stabilizes the leptin receptor through interaction with MAGEL2 as well as with RNF41 and USP8 (47).

Necdin may also enhance the cytoplasmic retention of proteins such as EID-1 and NEFA. Interestingly, cytoplasmic retention of EID-1 relieves its transcriptional repression, whereas enhanced cytoplasmic retention of NEFA potentiates its effect on the caffeine-evoked elevation of cytosolic Ca^2+^ levels (48).

Our study indicates that necdin may serve as a scaffold protein, facilitating SGT1-HSP90 chaperone machinery to stabilize the core circadian clock component BMAL1. Nevertheless, we cannot totally exclude the possibility that necdin may also regulate the BMAL1 protein level through other mechanisms. It will be intriguing to study whether necdin also affects the translation of BMAL1 protein.

### Post-translational regulation of BMAL1

The transcription of *Bmal1* is positively regulated by RORα, and antagonized by Rev-Erbα (10). In addition, BMAL1 protein is subjected to extensive post-translational modifications, such as acetylation, ubiquitination, sumoylation and phosphorylation (49–52). Interestingly, the acetylation and deacetylation of BMAL1 is catalyzed by its partner CLOCK and Sirt1, respectively. Although necdin is able to recruit Sirt1 to de-acetylate Foxo1 and p53, it is unlikely that this pathway explains the effect of necdin on BMAL1. First, BMAL1 acetylation does not affect its stability (53), whereas necdin positively regulates BMAL1 protein levels. Second, BMAL1 acetylation facilitates recruitment of CRY1 to CLOCK-BMAL1, thereby promoting transcriptional repression (53). In contradistinction, necdin correlates positively with the transcriptional activity of BMAL1.

### The effect of *necdin* deficiency on *Per2* expression

It should be noted that *Per2* mRNA level was only subtly downregulated in the livers from *necdin*^−p/+m^ mice, although BMAL1 protein levels were significantly damped. The oscillation of *Per2* in *necdin*-deficient mice may be driven by the system cues, such as body temperature and feeding. In a seminar study, Kornmann *et al.* repressed hepatic *Bmal1* transcription by overexpressing *Rev-erba* in the liver. Although this manipulation damped the rhythmic transcription of most genes, the expression of *Per2* oscillated robustly (54,55).

Consistent with the mRNA levels, the circadian rhythm of mPER2 from the liver nuclear extracts were also largely intact in *necdin*^−p/+m^ mice. PER1, PER2, CRY1 and CRY2 proteins form a repressor complex with a molecular mass of ∼2 MDa. Among them, PER2 may be the rate-limiting component for the formation of this repressor complex. Schibler *et al.* speculate that CRY proteins and PER1 may be produced in excess, and the proteins that are not incorporated into the repressosome complex may be rapidly degraded (56). Indeed, necdin-deficiency did not alter the pattern of mCRY2 in the liver nuclear extracts, in spite of the significant downregulation of Cry2 mRNA levels.

In conclusion, we have demonstrated that the PWS protein necdin regulates BMAL1 stability and circadian rhythm amplitudes through the SGT1-HSP90 pathway. Our data uncovered a possible mechanism underlying the sleep disorders observed in PWS patients, and further highlighted an important role for protein homeostasis in circadian control. It will be intriguing to identify co-chaperones/ chaperones that control other components of the circadian clock.

## Supplementary Material

gkaa601_Supplemental_File
