# Supplementary material for: Necdin regulates BMAL1 stability and circadian clock through SGT1-HSP90 chaperone machinery
Source: Nucleic Acids Res. 2020 Jul 15;48(14):7944–57. doi: 10.1093/nar/gkaa601 (PMC7430654; doi:10.1093/nar/gkaa601)
Supplement: gkaa601_Supplemental_File [file gkaa601_supplemental_file.pdf]

Supplemental data information

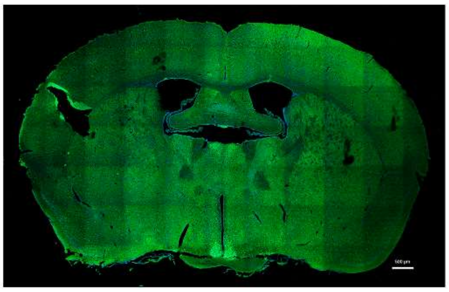

Figure S1. Expression of necdin (green) in the SCN as assayed by immunofluorescence staining. Scale bar, 500 μm.

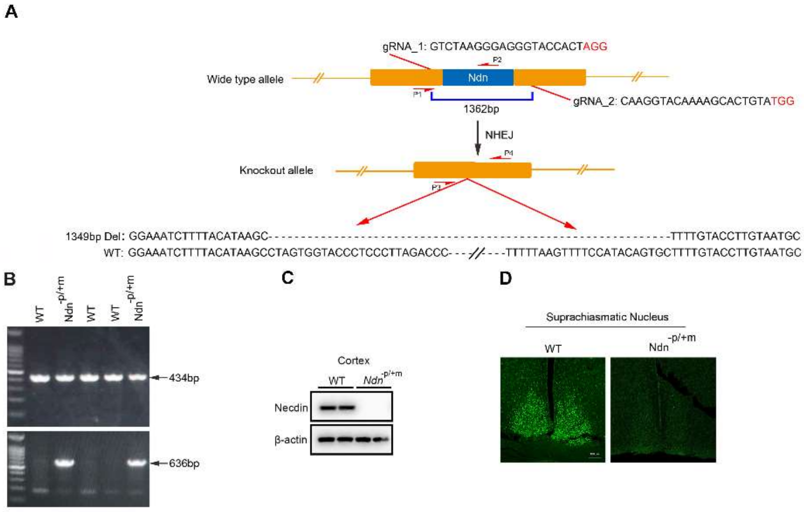

Figure S2. Generation of *necdin* knockout mice. **(A)** CRISPR-cas9 was used to generate *necdin*-null mice. Two gRNAs (gRNA_1 and gRNA_2) targeting the upstream and downstream regions of the *necdin* gene were injected with Cas9 mRNA into mouse oocytes, resulting in deletion of a 1349 bp fragment. Primers P1 and P2 were used to amplify the WT allele, whereas primers P3 and P4 were used to amplify the KO allele. **(B)** Genotyping by PCR. The PCR product for the WT allele is 434 bp, whereas that for the KO allele is 636 bp. **(C)** Necdin expression in the cortex of WT and *Ndn*$^{-p/+m}$ mice as assayed by Western blot. **(D)** Necdin expression in the suprachiasmatic nuclei of WT and *Ndn*$^{-p/+m}$ mice as assayed by immunofluorescence staining.

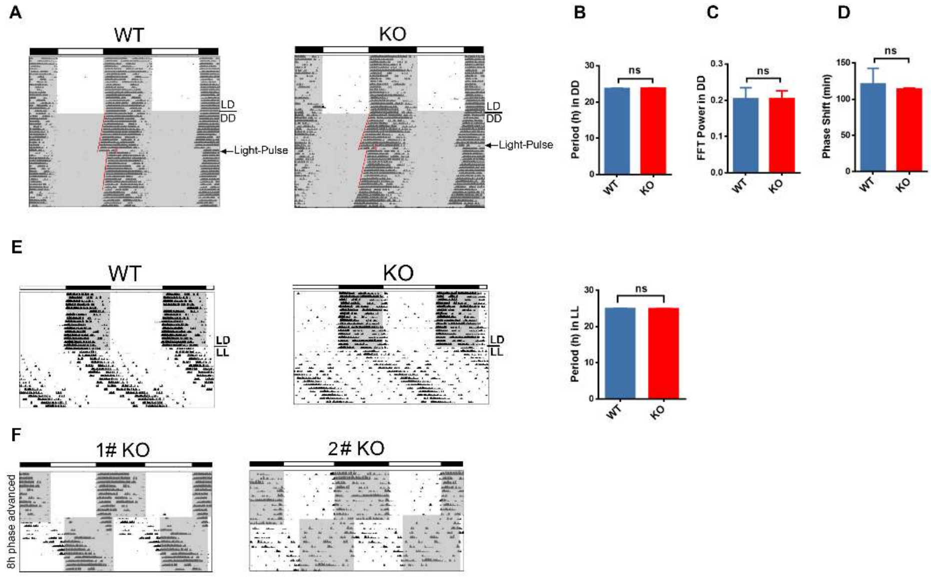

Figure S3. Wheel-running activities of WT and *necdin* KO mice. **(A)** Forty-eight-hour double plots are shown in which the recordings of each day are replicated and appear under those of the previous day. Animals were initially housed in 12:12-h light-dark (LD) conditions and then transferred to constant darkness (DD). The shift from LD to DD is indicated. A light pulse at CT16 during DD is also indicated. The timing of the LD cycle is indicated by the horizontal bar above each record and gray shading; dark bars and gray shadings indicate lights off, and open bars indicate lights on. Bin size is 5 min. **(B)-(C)** Statistics data of the free-running period (B) and FFT power (C) of WT and *Ndn* KO mice in DD. Data are presented as means ± s.e.m.; ns: no significance, $p > 0.05$, Student's *t*-test; WT, n = 6, KO, n=6. **(D)** Statistics data of the phase shift after a light pulse at CT16 of WT and *Ndn* KO mice in DD. Data are presented as means ± s.e.m.; ns: no significance, $p > 0.05$, Student's *t*-test; WT, n = 6, KO, n=6. **(E)** Representative double-plotted actograms and statistics data of the free-running period of activity of wild-type (WT) and *Ndn* KO mice in constant light (LL). Mice were housed under a 12 hr:12 hr light : dark (LD) cycle followed by LL conditions for 4 weeks. Data are presented as means ± s.e.m.; ns: no significance, $p > 0.05$, Student's t test; WT, n = 6, KO, n=6. **(F)** Jet lag treatment disrupted the behaviors in two *necdin* KO mice.

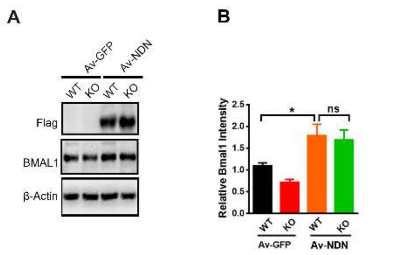

Figure S4. Necdin increases BMAL1 protein level in *necdin* KO MEF cells. Representative immunoblots (A) and statistics data of three independent experiments (B) from WT and *necdin* KO MEF cells infected with GFP or *NDN* adenovirus. Data are presented as means ± s.e.m.; *: $p < 0.05$, ns: no significance, unpaired *t*-test.

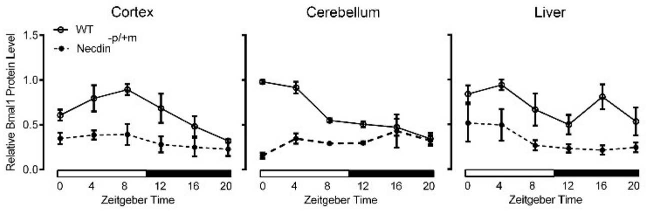

Figure S5. Quantification of BMAL1 protein level in the cortex, cerebellum and livers from WT and *necdin* KO mice. Data are presented as means ± s.e.m. n = 3 mice/genotype/time point.

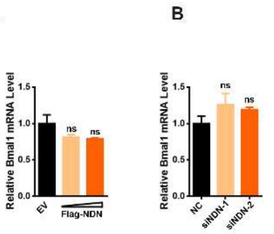

Figure S6. Necdin has no effect on Bmal1 mRNA level. Overexpression (A) or depletion (B) of necdin did not influence the transcription of *Bmal1* as assayed by quantitative PCR. EV, empty vector; NC, control siRNA. Data are presented as means ± s.e.m.; ns: no significance, unpaired *t*-test.

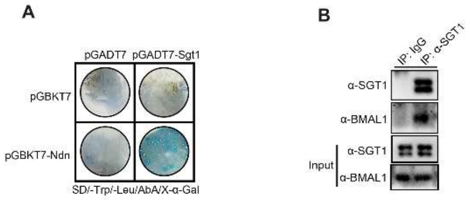

Figure S7. Interaction of necdin/SGT1 and SGT1/BMAL1. **(A)** Interaction of necdin and SGT1 as assayed by a yeast two hybrid assay. The term pGBKT7-Ndn denotes necdin fused with a DNA-binding domain; pGADT7-Sgt1 denotes SGT1 fused with an activation domain. Only yeast colonies co-transformed with pGADT7-Sgt1 and pGBKT7-Ndn grow and turn blue on a SD/-Leu/-Trp/AbA/X-α-Gal plate. **(B)** Interaction of SGT1 and BMAL1 as assayed by a co-IP assay in U2OS cells. Cell lysate was immunoprecipitated with an antibody against SGT1 or control IgG, and the immune complex was blotted with antibodies against SGT1 or BMAL1, respectively.

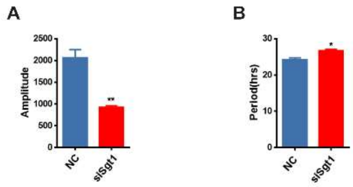

Figure S8. Depletion of SGT1 significantly affects circadian rhythms in U2OS cells. Amplitudes (A) or periods (B) obtained from Bmal1:luciferase U2OS cells transfected with control siRNA (NC) or Sgt1 siRNA. Data are presented as means ± s.e.m.; *: $p < 0.05$; **: $p < 0.01$, Student's *t*-test, n = 6.
